# Modulation of Cardiac Ventricular Excitability by GLP-1 (Glucagon-Like Peptide-1)

**DOI:** 10.1161/CIRCEP.118.006740

**Published:** 2018-10-11

**Authors:** Richard Ang, Svetlana Mastitskaya, Patrick S. Hosford, Marina Basalay, Mark Specterman, Qadeer Aziz, Yiwen Li, Michele Orini, Peter Taggart, Pier D. Lambiase, Andrey Gourine, Andrew Tinker, Alexander V. Gourine

**Affiliations:** 1 Centre for Cardiovascular and Metabolic Neuroscience, Neuroscience, Physiology & Pharmacology, University College London, United Kingdom (R.A., S.M., P.S.H., M.B., A.V.G.); 2 Institute of Cardiovascular Science, University College London, United Kingdom (M.O., P.T., P.D.L.); 3 William Harvey Research Institute, Barts and the London School of Medicine and Dentistry, London, United Kingdom (R.A., M.S., Q.A., Y.L., A.T.).; 4 Division of Cardiology, Karolinska Institute, Stockholm, Sweden (A.G.).

**Keywords:** action potentials, cardiovascular diseases, diabetes mellitus, glucagon like peptide-1, nitric oxide

## Abstract

**Background:**

Glucagon-like peptide-1 receptor (GLP-1R) agonists improve cardiovascular outcomes in patients with type 2 diabetes mellitus. However, systemic actions of these agents cause sympathetic activation, which is generally considered to be detrimental in cardiovascular disease. Despite significant research interest in cardiovascular biology of GLP-1, the presence of GLP-1R in ventricular cardiomyocytes remains a controversial issue, and the effects of this peptide on the electrical properties of intact ventricular myocardium are unknown. We sought to determine the effects of GLP-1R agonist exendin-4 (Ex4) on ventricular action potential duration (APD) and susceptibility to ventricular arrhythmia in the rat heart in vivo and ex vivo.

**Methods:**

Ventricular monophasic action potentials were recorded in anaesthetized (urethane) rats in vivo and isolated perfused rat hearts during sinus rhythm and ventricular pacing.

**Results:**

In vivo, systemic administration of Ex4 (5 μg/kg intravenously) increased heart rate, and this effect was abolished by β-adrenoceptor blockade. Despite causing sympathetic activation, Ex4 increased APD at 90% repolarization during ventricular pacing by 7% (*P*=0.044; n=6) and reversed the effect of β-adrenoceptor agonist dobutamine on APD at 90% repolarization. In isolated perfused hearts, Ex4 (3 nmol/L) increased APD at 90% repolarization by 14% (*P*=0.015; n=6) with no effect on heart rate. Ex4 also reduced ventricular arrhythmia inducibility in conditions of β-adrenoceptor stimulation with isoproterenol. Ex4 effects on APD and ventricular arrhythmia susceptibility were prevented in conditions of muscarinic receptor blockade or inhibition of nitric oxide synthase.

**Conclusions:**

These data demonstrate that GLP-1R activation effectively opposes the effects of β-adrenoceptor stimulation on cardiac ventricular excitability and reduces ventricular arrhythmic potential. The effect of GLP-1R activation on the ventricular myocardium is indirect, mediated by acetylcholine and nitric oxide and, therefore, can be explained by stimulation of cardiac parasympathetic (vagal) neurons.

**WHAT IS KNOWN?**

- GLP-1 (glucagon-like peptide-1) receptor agonists improve cardiovascular outcomes in patients with type 2 diabetes mellitus.
- Despite significant research interest in cardiovascular biology of GLP-1, the presence of GLP-1 receptor in ventricular cardiomyocytes remains a controversial issue, and the effects of this peptide on the electrical properties of intact ventricular myocardium are unknown.

**WHAT THE STUDY ADDS?**

- GLP-1 receptor activation opposes sympathetic effects on cardiac ventricular excitability and reduces ventricular arrhythmic potential.
- These effects are indirect, mediated by acetylcholine and nitric oxide, and can be explained by facilitated release of these signaling molecules from the ventricular terminals of cardiac vagal neurons.

GLP-1 (glucagon-like peptide-1) is an incretin hormone produced predominantly by L-cells of the gut in response to the ingestion of food. GLP-1 plays an important role in the physiological control of glucose metabolism and energy homeostasis.^1^ GLP-1 receptor (GLP-1R) agonists are currently in clinical use for the treatment of diabetes mellitus and obesity.^2^ Because of significant beneficial effects of GLP-1R agonists on the cardiovascular system (including anti-inflammatory, antiatherogenic, positive inotropic, glucose uptake stimulating, and vasodilatory effects),^2^ GLP-1 has become the most extensively studied incretin. Based on significant preclinical data, large-scale clinical trials were initiated to test the efficacy of GLP-1R agonists on cardiovascular outcomes in various patient cohorts. A recent influential study conducted in ≈10 000 patients with type 2 diabetes mellitus demonstrated significant reduction in frequency of adverse cardiovascular events and death from cardiovascular causes by treatment with GLP-1 analogue liraglutide.^3^

GLP-1 acutely increases heart rate (HR), and this effect of GLP-1 on nodal tissue appears to be indirect, mediated via increased activity of the sympathetic nervous system.^4,5^ Despite triggering sympathetic activation and increases in HR (which would be expected to increase myocardial oxygen demands, worsen myocardial injury, and promote arrhythmias), GLP-1R agonists have been found to effectively protect cardiomyocytes against ischemia/reperfusion injury in animal models^2,3,6,7^ and in humans.^8^ There is also evidence that GLP-1 may function as a humoral factor that mediates the innate mechanisms of remote ischemic preconditioning cardioprotection.^9,10^ However, despite significant research interest in cardiovascular biology of GLP-1, the presence of GLP-1R in ventricular cardiomyocytes remains a controversial issue,^11,12^ and the effects of the peptide on the electrical properties of intact ventricular myocardium and in vivo remain unknown. This study conducted in experimental animals (rats) investigated the effect of GLP-1R activation on the ventricular electrophysiological properties in vivo and in isolated heart preparations. We hypothesized that this approach would also reveal the presence of functional GLP-1R in ventricular myocytes if (1) the actions of GLP-1R agonist are associated with significant changes in the electrical properties of the ventricular myocardium, (2) these effects of GLP-1R agonist are preserved in isolated hearts, and (3) the effects of GLP-1R agonist are preserved in conditions when common autonomic effector signaling mechanisms are blocked pharmacologically.

## Methods

The data, analytic methods, and study materials are available to other researchers for purposes of reproducing the results or replicating the procedures. All experiments were performed in accordance with the European Commission Directive 2010/63/EU (European Convention for the Protection of Vertebrate Animals used for Experimental and Other Scientific Purposes) and the UK Home Office Scientific Procedures Act (1986) with project approval from the respective Institutional Animal Welfare and Ethical Review Bodies.

### Experiments In Vivo

Adult male Sprague-Dawley rats (250–340 g) were anaesthetized with urethane (1.3 g/kg intraperitonially). The right femoral artery and vein were cannulated for measurement of the arterial blood pressure and administration of pharmacological agents, respectively. The trachea was cannulated, and the animal was mechanically ventilated with room air using a positive pressure ventilator (tidal volume ≈1 mL per 100 g of body weight, ventilator frequency 60 strokes/min). Adequate level of anesthesia was ensured by monitoring stability of arterial blood pressure and HR and the absence of a withdrawal response to a paw pinch. Po_2_, Pco_2_, and pH of the arterial blood were measured regularly, and (if required) ventilation was adjusted to maintain these variables within the physiological ranges. The body temperature was maintained at 37°C±0.5°C with a servo-controlled heating pad.

The heart was exposed via a left thoracotomy, and a bipolar silver pacing electrode was fixed to the right ventricular apex using a 4-0 monofilament polypropylene suture. Pacing stimuli were delivered (S88-Grass stimulator) at twice the diastolic threshold. Ventricular monophasic action potential (MAP) was recorded using a custom-made suction electrode placed directly onto the epicardial surface of the left ventricle. Arterial blood pressure, a standard lead II ECG, and ventricular MAP signals were recorded using Power1401 interface and analyzed offline using Spike2 software (Cambridge Electronic Design Ltd).

Action potential duration (APD) at 90% repolarization (APD_90_) was determined in sinus rhythm and during ventricular pacing at ≈500 bpm to allow APD_90_ comparisons at fixed HR. Values of HR and PR interval were averaged from 10 consecutive beats during steady state for each condition. Pacing was performed for 1 minute, and APD_90_ measurements were taken after averaging of MAP in the last 10 seconds when the recording had stabilized. The MAP amplitude was measured as the distance from the diastolic baseline to the crest of the MAP plateau phase, not the peak of the upstroke. The amplitude of the recorded MAP signal was generally in the range of 5 to 15 mV. The MAP duration was measured as the interval from the steepest part of the MAP upstroke (or the intrinsic deflection, if discernible) to the 90% repolarization level along a line horizontal to the diastolic baseline, as described.^13^

**Figure 1.**
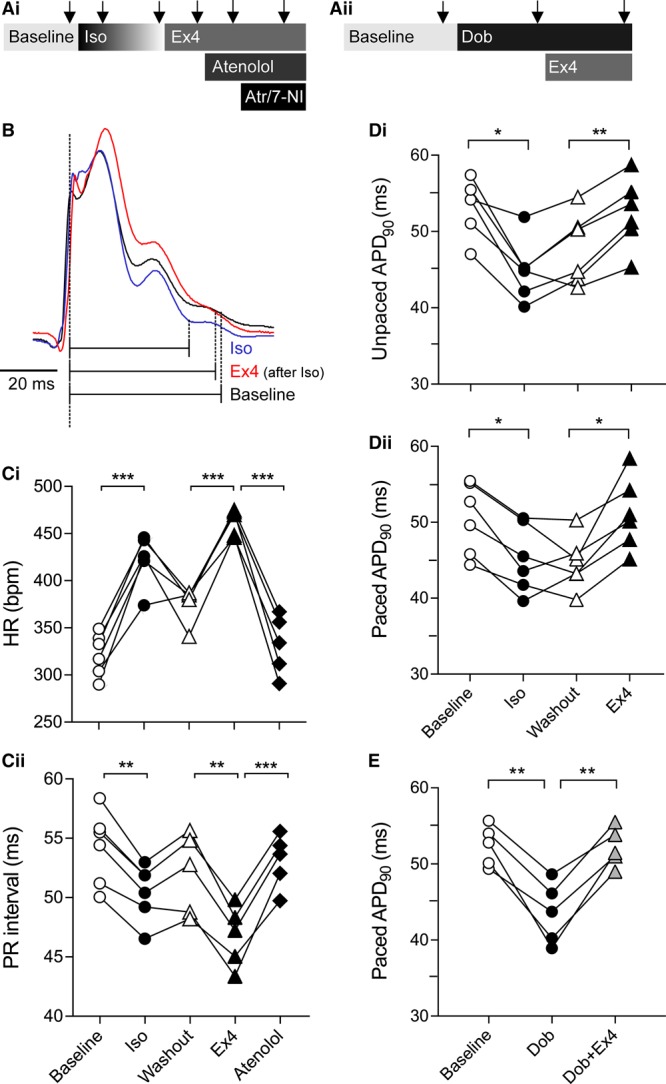
**Effects of GLP-1 (glucagon-like peptide-1) receptor activation on heart rate and electrical properties of the ventricular myocardium in vivo.A**, Experimental protocols of the in vivo experiments. Arrows depict time points when physiological measurements were taken; (**B**) Representative recordings of monophasic action potential illustrating the effects of isoproterenol (Iso, 2 μg/kg intravenously) and exendin-4 (Ex4, 5 μg/kg intravenously) on action potential duration at 90% repolarization (APD_90_); (**C**) Effects of Iso and Ex4 on heart rate (HR, **Ci**) and PR interval (**Cii**); (**D**) Summary data showing the effects of Iso and Ex4 on APD_90_ during sinus rhythm (**Di**) and during fixed pacing (**Dii**); (**E**) Effects of dobutamine (Dob, 1.5 mg/kg intraperitonially) and coadministration of Dob (1.5 mg/kg intraperitonially) and Ex4 (5 μg/kg intravenously) on APD_90_ in fixed HR conditions. *P<0.05, **P<0.01, ***P<0.001.

In a group of 6 rats, MAP morphology was sequentially assessed: (1) at baseline; (2) in conditions of systemic β-adrenoceptor (β-AR) stimulation with isoproterenol (Iso; 2 μg/kg intravenously); (3) following washout of Iso effect (≈10 minutes after administration of Iso); (4) in conditions of systemic administration of GLP-1R agonist exendin-4 (Ex4; 5 μg/kg intravenously), and then following autonomic blockade with (5) β-AR blocker atenolol (2 mg/kg intravenously); and finally (6) muscarinic receptor antagonist atropine (2 mg/kg intravenously; n=3) or neuronal nitric oxide (NO) synthase inhibitor 7-nitroindazole (25 mg/kg intraperitonially; n=3) administration (Figure 1Ai). The 5 μg/kg dose of Ex4 was chosen based on the results of published studies that demonstrated potent cardioprotection of GLP-1R activation in vivo.^9,14^

In a separate group of 5 rats, MAP was sequentially assessed: (1) at baseline, (2) in conditions of systemic administration of a longer acting β-AR agonist dobutamine (1.5 mg/kg intraperitonially), and (3) in conditions of systemic coadministration of dobutamine (1.5 mg/kg intraperitonially) and Ex4 (5 μg/kg intravenously; Figure 1Aii).

### Experiments in Isolated Heart Preparation

Sprague-Dawley rats (125–150 g) were terminally anaesthetized with pentobarbital sodium (100 mg/kg intraperitonially). Hearts were excised after thoracotomy, placed in ice-cold Krebs solution (NaCl 118 mmol/L, KCl 4.75 mmol/L, MgSO_4_ 1.19 mmol/L, NaHCO_3_ 25 mmol/L, KH_2_PO_4_ 1.19 mmol/L, d-glucose 5 mmol/L, CaCl_2_ 1.4 mmol/L, and pyruvate 2 mmol/L), cannulated via the aorta, and mounted onto a Langendorff apparatus to establish retrograde perfusion via the ascending aorta with oxygenated Krebs solution at a constant flow of 2 mL/min. Ventricular MAP was recorded using Franz contact electrode (ADInstruments) placed directly onto the epicardial surface of the left ventricle. The temperature of the preparation was maintained at 37.0°C±0.5°C. ECG and MAP signals were recorded using Power1401 interface and analyzed offline using Spike2 software. Experimental protocols commenced after a 15-minute stabilization period. To deliver ventricular pacing, a custom-made bipolar silver electrode was placed onto the apex of the right ventricle. Using a constant current stimulator (Digitimer DS7A), a train of 15 pulses was applied (S1, 0.5 mA; cycle length, 110 ms), followed by an incrementally shortened period before application of an extra single pulse (S2) until failure of ventricular capture. APD_90_ was determined after averaging of the last 3 paced beats of all S1 pacing trains. To assess susceptibility to ventricular arrhythmia, the number of ventricular extrasystoles induced after each S2 pulse was counted with ≥4 ventricular extrasystoles defined as ventricular tachycardia.

**Figure 2.**
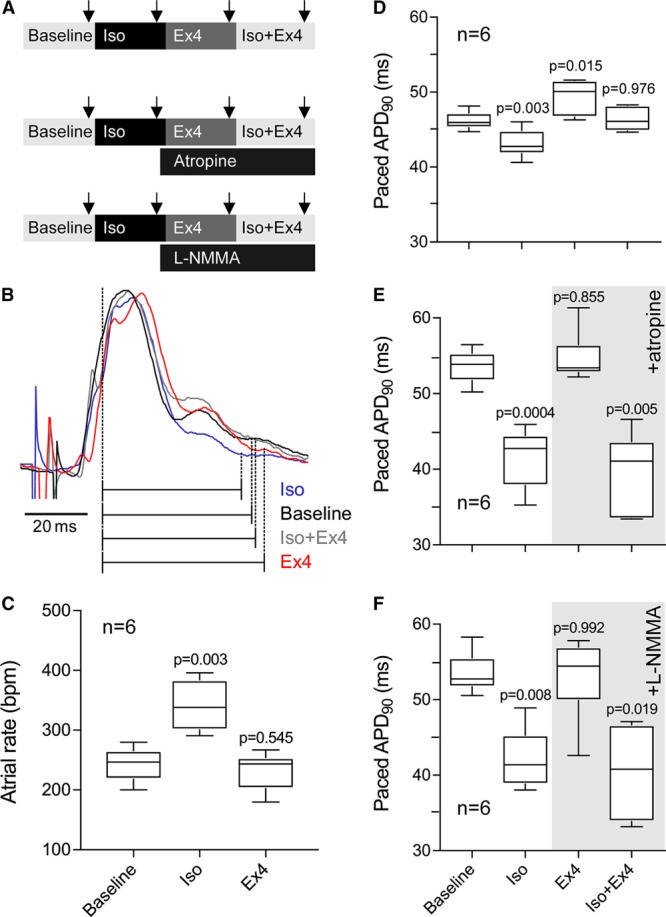
**Effects of GLP-1 (glucagon-like peptide-1) receptor activation on atrial rate and electrical properties of the ventricular myocardium ex vivo.A**, Experimental protocols in isolated perfused hearts. Arrows depict time points when physiological measurements were taken; (**B**) Representative recordings of monophasic action potential illustrating the effects of isoproterenol (Iso; 100 nmol/L), exendin-4 (Ex4; 3 nmol/L), and coadministration of Iso (100 nmol/L) and Ex4 (3 nmol/L) on paced action potential duration at 90% repolarization (APD_90_) in isolated hearts; (**C**) Effects of Iso and Ex4 on atrial rate in isolated hearts; Summary data showing the effects of Iso, Ex4, and coadministration of Iso and Ex4 on paced APD_90_ in the absence (**D**) and presence of atropine (1 μmol/L, **E**) or L-N^G^-monomethyl arginine (L-NMMA, 100 μmol/L, **F**). *P* values indicate the level of significance compared with baseline condition.

In a group of 6 hearts, APD_90_ and ventricular arrhythmia susceptibility were assessed: (1) at baseline, (2) in conditions of β-AR stimulation with Iso (100 nmol/L), (3) after administration of Ex4 (3 nmol/L), and finally, (4) in conditions of Iso (100 nmol/L) and Ex4 (3 nmol/L) coadministration (Figure 2A). For these experiments, the 3 nmol/L dose of Ex4 was chosen based on the results of published studies which demonstrated potent cardioprotection of GLP-1R activation in isolated rodent hearts.^15–17^

In 2 separate experimental groups, the effects of Ex4 were also assessed in conditions of muscarinic receptor blockade with atropine (1 μmol/L; n=6) and NO synthase inhibition with L-N^G^-monomethyl arginine (100 μmol/L; n=6; Figure 2A).

### Statistical Analysis

Data are reported as individual values, means±SEM, or as box and whiskers plots. Groups were compared by repeated measures ANOVA with Greenhouse-Geisser correction followed by Tukey post hoc test. Categorical data were compared by χ^2^test and Fisher exact test. Differences between the groups or treatments with *P*<0.05 were considered to be significant. Statistical analysis was performed using GraphPad Prism 7 software. The data that support the findings of this study are available from the corresponding authors on request.

## Results

### Effects of GLP-1R Agonist Ex4 on HR and Electrical Properties of the Ventricular Myocardium In Vivo

Because systemic actions of GLP-1R agonists increase HR indirectly via triggering sympathetic activation, we first compared the effects of Ex4 to that of β-AR agonist Iso. Systemic administration of Iso (2 μg/kg intravenously) increased HR from 322±9 to 423±11 bpm (*P*<0.001; n=6) and reduced PR interval from 54.2±1.3 to 50.5±1.0 ms (*P*=0.003; Figure 1C). Iso also reduced APD_90_ (from 53.2±1.5 to 44.9±1.6 ms; *P*=0.022; Figure 1B and 1Di).

Ex4 (5 μg/kg intravenously, administered after allowing the effect of Iso to plateau during washout) increased HR (from 367±9 to 460±6 bpm; *P*<0.001) and reduced PR interval (from 52.5±1.3 to 47.3±1.1 ms; *P*=0.001; Figure 1C). Because HR and PR interval at washout were slightly different when compared with the baseline (reflecting incomplete Iso washout), the magnitude of Ex4 effect on these variables is likely to be somewhat underestimated. Importantly, in contrast to the effect of Iso, Ex4 prolonged ventricular APD_90_ (52.4±1.9 versus 47.8±1.9 ms; *P*=0.006; Figure 1B and 1Di). Administration of β-AR antagonist atenolol reversed the effects of Ex4 on HR and PR interval (Figure 1C) but had no effect on APD_90_ changes induced by Ex4 (*P*=0.285). Both atropine and 7-nitroindazole administered after Ex4, reduced APD_90_ by the same degree (to 40.1±0.7 and 41.5±2.9 ms, respectively; *P*=0.002 compared with Ex4).

To separate the effects of GLP-1R activation on the electrical properties of the ventricular myocardium from that confounded by GLP-1R–mediated sympathetic activation and increases in HR, ventricular MAP was recorded in conditions of fixed ventricular pacing. With HR fixed between the conditions, Iso reduced APD_90_ from 50.5±1.9 to 45.2±1.8 ms (*P*=0.014; Figure 1Dii). Subsequent administration of Ex4 resulted in a significant prolongation of paced APD_90_ from 44.6±1.4 to 51.1±1.9 ms (*P*=0.044; Figure 1Dii). Atenolol had no effect on paced APD_90_ in conditions of systemic Ex4 action (47.0±1.4 ms; *P*=0.314); however, the effect of Ex4 on APD_90_ was reversed by atropine and 7-nitroindazole (to 42.9±2.2 and 38.4±1.4 ms, respectively; *P*=0.02).

In a separate experiment, systemic administration of dobutamine (1.5 mg/kg intraperitonially) reduced paced APD_90_ from 52.4±1.2 to 43.5±1.8 ms (*P*=0.008; n=5). Ex4 effectively reversed the effect of β-AR stimulation on paced APD_90_ (increase from 43.5±1.8 to 52.2±1.1 ms; *P*=0.005 and *P*=0.952 compared with baseline; Figure 1E).

### Effects of GLP-1R Agonist Ex4 on Atrial Rate and Electrical Properties of the Ventricular Myocardium Ex Vivo

In the isolated heart preparation, Iso (100 nmol/L) increased atrial rate (from 243±12 to 341±17 bpm; *P*=0.003; n=6) while Ex4 (3 nmol/L) had no effect (232±13 versus 243±12 bpm; *P*=0.545; Figure 2C).

Iso reduced paced APD_90_ from 46.2±0.5 to 43.2±0.8 ms (*P*=0.003). Ex4 had an opposite effect and increased APD_90_ to 49.4±1.0 ms (*P*=0.015 compared with baseline; Figure 2D). Combined application of Iso and Ex4 had no effect on APD_90_ when compared with baseline (46.4±0.6 versus 46.2±0.5 ms; *P*=0.976; Figure 2D). The effects of Ex4 on APD_90_ in the absence and presence of β-AR agonist Iso were prevented by atropine (1 μmol/L; n=6) and NO synthase inhibitor L-N^G^-monomethyl arginine (100 μmol/L; n=6; Figure 2E and 2F).

### Effect of GLP-1R Agonist Ex4 on the Ventricular Arrhythmia Potential

**Figure 3.**
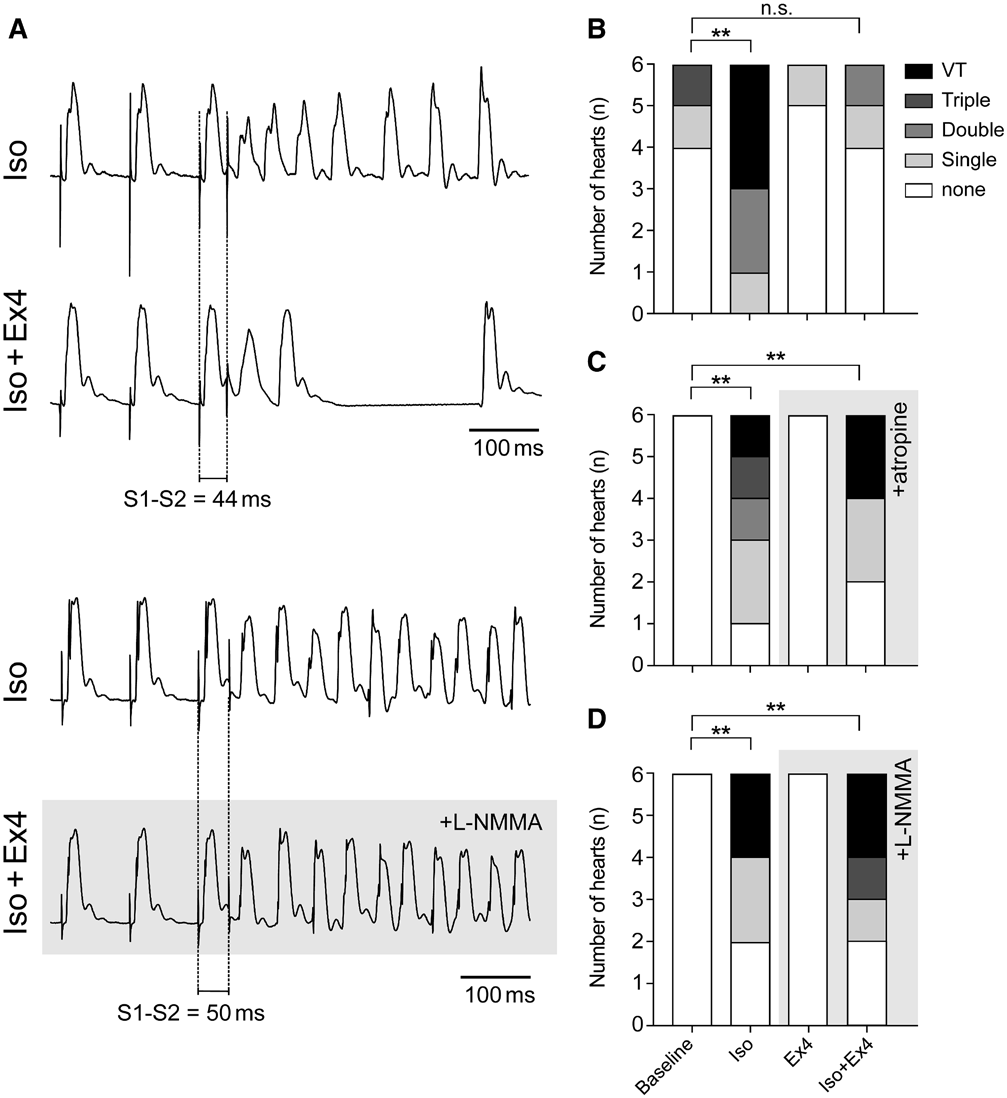
**Effect of GLP-1 (glucagon-like peptide-1) receptor activation on the ventricular arrhythmia potential.A**, Representative recordings of restitution pacing to induce ventricular tachycardia (V_T_) in the presence of isoproterenol (Iso, 100 nmol/L). Coadministration of exendin-4 (Ex4, 3 nmol/L) protects against V_T_ (**top**), and this effect of Ex4 is abolished by nitric oxide synthase blockade with L-N^G^-monomethyl arginine (L-NMMA, 100 μmol/L; **bottom**); Effects of Iso (100 nmol/L), Ex4 (3 nmol/L), and coadministration of Iso (100 nmol/L) and Ex4 (3 nmol/L) on V_T_ inducibility in the absence (**B**) and presence of atropine (1 μmol/L, **C**) or L-NMMA (100 μmol/L, **D**). **P<0.01, n.s. indicates not significant.

In isolated heart preparation, Iso increased ventricular tachycardia inducibility during restitution pacing from 0/18 hearts at baseline to 6/18 (*P*=0.007 χ^2^, *P*=0.019 Fisher exact test; Figure 3). In 6 hearts, coadministration of Ex4 with Iso rendered ventricular tachycardia noninducible (0/6 Ex4+Iso versus 3/6 Iso alone, *P*=0.046 χ^2^, *P*=0.182 Fisher exact test; Figure 3A and 3B). In 12 hearts, treatment with either atropine or L-N^G^-monomethyl arginine abolished the effects of Ex4 (Figure 3C and 3D).

## Discussion

In this study, we investigated the effects of a prototypical GLP-1R agonist Ex4 on tissue-level ventricular electrophysiology in the rat heart. APD was assessed from MAPs recorded during sinus rhythm and during ventricular pacing. Consistent with the previously reported data,^5^ systemic actions of Ex4 in vivo increased HR, and this effect was abolished by β-AR blockade. In contrast to the effect of Iso, Ex4 prolonged APD. This effect was more pronounced in fixed HR conditions where Ex4 markedly increased APD and effectively counteracted the effect of pharmacological β-AR stimulation on the heart. Similarly, in isolated heart preparation, Ex4 prolonged APD and prevented the effect of Iso on APD, but had no effect on HR. Furthermore, Ex4 decreased arrhythmia inducibility in conditions of simulated sympathetic activation mimicked by application of β-AR agonist Iso.

GLP-1R is coupled to the stimulatory G-proteins, and the actions of GLP-1 or GLP-1 analogues lead to activation of protein kinase A signaling pathway,^18^ similar to that recruited by β-AR stimulation. However, the effect of Ex4 on APD is opposite to that of β-AR agonists, and this is consistent with the current view that GLP-1R are not expressed by ventricular myocytes.^11^ Thus, the effects of Ex4 on ventricular electrical properties are likely to be mediated by GLP-1Rs expressed by tissues extrinsic to cardiac myocytes.

That the effect of Ex4 on ventricular APD is preserved in isolated perfused heart preparation suggests that this GLP-1R agonist may act on either coronary blood vessels (vascular endothelium or smooth muscle) or the nerves which originate from the intrinsic cardiac ganglia and provide innervation of the ventricular system.^19,20^ Identification of the exact mechanism would require new approaches, such as conditional deletion of GLP-1Rs from the target tissue using mouse models. However, our pharmacological analysis suggests that Ex4 is likely acting on parasympathetic (vagal) neurons of the intrinsic cardiac ganglia which release acetylcholine and NO from their ventricular terminals. Indeed, the effects of Ex4 on APD were reversed or prevented by either muscarinic blockade or inhibition of the neuronal NO synthase. These effects are consistent with vagal modulation of ventricular excitability mediated by the actions of acetylcholine and NO.^21,22^

The exact mechanisms of GLP-1R–mediated protective effects on ventricular arrhythmic potential remain to be determined. These may involve modulation of reentrant circuit path length through changes in cellular refractoriness. However, we have not examined changes in conduction velocity or changes in calcium handling that might underlie changes in abnormal impulse formation through delayed afterdepolarizations.

Interestingly, certain mutations, for example, in the gene encoding K_v_11.1 voltage-gated K^+^ channels cause long-QT syndrome 2 and simultaneously increase the production of GLP-1 by intestinal L cells.^23^ The data obtained in this study suggest that increased circulating GLP-1 in long-QT syndrome 2 may potentially be antiarrhythmic. However, it could also worsen the QT prolongation under conditions of increased sympathetic drive, such as during exercise.

The use of GLP-1R agonists has been an important advance in the care of patients with type 2 diabetes mellitus.^1^ Initially, there were concerns that these agents might worsen cardiovascular outcomes because of sympathetic activation.^24^ However, there are no such indications and genomic studies support a clear negative association with the severity of cardiovascular disease.^25^ Furthermore, clinical studies have shown beneficial effects of GLP-1R agonists on cardiovascular disease markers, including atherogenic marker apolipoprotein B.^26^ This study reveals a potential antiarrhythmic effect of GLP-1R activation, consistent with previously described strong cardioprotective effects of GLP-1.^2,6–9^ One potential complication with the use of the existing drugs is that they may increase sympathetic drive, which is generally considered to be maladaptive and detrimental, contributing to the development of cardiovascular disease. Thus, it would be important to determine the exact site of GLP-1 action on the heart and then try to target these specific receptor populations.

Acknowledged study limitations are related to the marked differences in action potential morphology between rodent heart and that of larger mammals, including humans, and potential species-specific differences in GLP-1R expression in the heart. The first limitation can potentially be addressed by studying the effect of GLP-1R agonists in preparations of human ventricular myocytes (eg, in organotypic cultured human cardiac slices),^27^ yet, these preparations might be lacking key components, including intact cardiac vagal neurons and their ventricular projections, which (as the data of this study suggest) mediate the effects of GLP-1 on the electrical properties of the ventricular myocardium. Regarding potential species-specific differences in GLP-1R expression in the heart, earlier data obtained in rodents and primates largely agree that GLP-1Rs are predominantly expressed in the atria.^11,28,29^ The most recent report concluded that human cardiac ventricles do express GLP-1Rs, but the identity of the ventricular cell types expressing the receptor remains unknown.^12^ Ventricular GLP-1R mRNA transcripts detected in the RNA isolated from the ventricular tissue^12^ may reflect the expression of the receptor in cardiac nerve fibers which provide functional innervation of the ventricles.^19,22,30–32^

Taken together, the data obtained demonstrate that GLP-1R activation effectively opposes the effect of β-AR stimulation on cardiac APD and reduces the ventricular arrhythmic potential in conditions of sympathetic activation. The effects of GLP-1R activation on ventricular electrical properties appear to be indirect, mediated by acetylcholine and NO, and, therefore, can be explained by recruitment of cardiac vagal neuron activity.

## Sources of Funding

This work was supported by the British Heart Foundation (RG/14/4/30736 to Drs Gourine and Tinker; RG/15/15/31742 to Dr Tinker), European Union’s Horizon 2020 research and innovation programme (Marie Skłodowska-Curie Grant No. 654691 to Dr Mastitskaya), Medical Research Council (MR/N02589X/1), and The National Institute for Health Research Barts Cardiovascular Biomedical Research Centre. Dr Gourine is a Wellcome Trust Senior Research Fellow (Ref: 200893).

## Disclosures

None.

## Supplementary Material
