# Supplementary figures and images for: Modulation of Cardiac Ventricular Excitability by GLP-1 (Glucagon-Like Peptide-1)
Source: Circ Arrhythm Electrophysiol. 2018 Oct 11;11(10):e006740. doi: 10.1161/CIRCEP.118.006740 (PMC6553567; doi:10.1161/CIRCEP.118.006740)

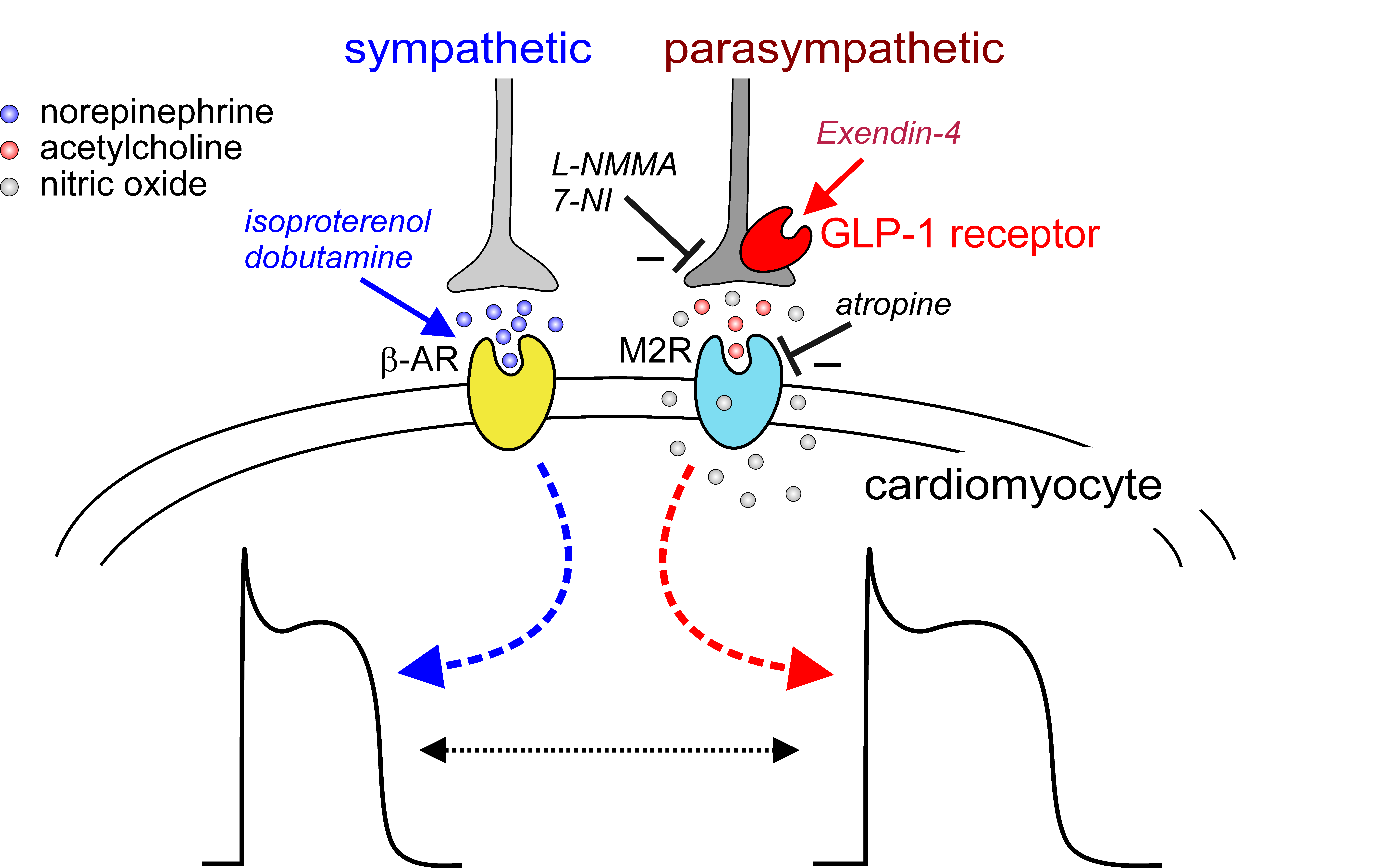

Supplement: Supplementary file 1 [file hae-11-e006740-s001.tif]
